# A multimodal *in vitro* approach to assess the safety of oral care products using 2D and 3D cellular models

**DOI:** 10.3389/ftox.2024.1474583

**Published:** 2024-11-06

**Authors:** S. Marceli Leano, Wanderson De Souza, Rodrigo De Vecchi, Amanda Lopes, Tatiana Deliberador, Jose M. Granjeiro

**Affiliations:** ^1^ Divisão de Metrologia em Biologia, Diretoria de Metrologia Científica, Industrial e Tecnologia, Instituto Nacional de Metrologia, Qualidade e Tecnologia (INMETRO), Duque de Caxias-RJ, Brazil; ^2^ Programa de Pós-Graduação em Biotecnologia, Instituto Nacional de Metrologia, Qualidade e Tecnologia (INMETRO), Rio de Janeiro-RJ, Brazil; ^3^ Episkin, Rio de Janeiro-RJ, Brazil; ^4^ Instituto Latino Americano de Pesquisa e Ensino Odontológico—ILAPEO, Curitiba-PR, Brazil

**Keywords:** toxicity, sodium perborate, oral care products, monolayer, three-dimensional culture, reconstructed human epidermis (RhE)

## Abstract

**Introduction:**

Periodontitis, affecting approximately 3.9 billion individuals globally, significantly impacts quality of life and has raised interest in its potential systemic effects. Sodium perborate, a common component in oral care products for biofilm control, is widely used, though concerns about its safety persist. This study aimed to evaluate the *in vitro* toxicity of six commercial oral care products and varying concentrations of sodium perborate, utilizing human gingival fibroblasts (HGF) and keratinocytes (HaCat) as cell models.

**Methods:**

Experiments were performed in both 2D monolayer and 3D cultures using MTT and electrical impedance assays, adhering to the manufacturer’s recommended exposure time of 30–60 s for product testing. For the reconstructed epidermis model, a prolonged exposure time of 42 min was applied, following the Organization for Economic Cooperation and Development (OECD) Test Guideline 439.

**Results:**

Results indicated that all products and sodium perborate at 1 mg/mL were cytotoxic in monolayer cultures. However, at concentrations relevant to commercial formulations (0.06 mg/mL sodium perborate), no significant toxicity was observed. In contrast, the 3D culture models, including spheroids and reconstructed epidermis, exhibited minimal to no cytotoxic effects for the commercial products, with sodium perborate showing no significant toxicity below 0.1 mg/mL. The reconstructed epidermis model, used as surrogate for oral mucosa, further confirmed that the products were non-irritating, in compliance with OECD TG 439 standards.

**Discussion:**

This study highlights the importance of considering exposure time, dosage, and cellular model when assessing the safety of oral care products. While 2D models are useful for preliminary screenings, 3D models provide a more physiologically relevant assessment, emphasizing the need for robust testing protocols to ensure product safety.

## 1 Introduction

Designated as a pandemic by the U.S. Centers for Disease Control and Prevention, periodontitis impacts 3.9 billion individuals worldwide (Albeshr et al., 2021). Studies have indicated a higher prevalence of periodontitis due to individual causal factors (Nazir, 2017) such as genetic and epigenetic susceptibility, lifestyle factors (Chapple et al., 2017) and various systemic diseases (Kuo et al., 2008) such as osteoporosis, atherosclerosis or diabetes (Di Stefano et al., 2022). Significant advancements in oral healthcare have emerged in recent years, leading to earlier disease diagnosis and a heightened emphasis on preventive strategies (Di Stefano et al., 2022; Revilla-León et al., 2022). Concurrently, improvements in techniques and materials have broadened dental expertise, contributing to developing more effective and long-lasting oral care products (Lin et al., 2019).

Oral care products like mouthwashes containing chlorhexidine (Weber et al., 2023), antibiofilm agents (Aspinall et al., 2021), carbamide peroxide, triclosan, essential oils (Yaghmoor et al., 2024) and sodium perborate are frequently used before surgery and dental procedures due to their antimicrobial properties (Hossainian et al., 2011) Sodium perborate is especially prevalent due to its benefits, including its effective bleaching properties, its role as an antimicrobial agent, and its capacity to release oxygen, which aids in wound healing and tissue regeneration (Kahler, 2022). However, concerns about its safety have arisen in studies, prompting uncertainty in the industry and among regulators about product registration, necessitating further tests and safety evaluations (Montaner et al., 2024).

Research indicates potential health risks with sodium perborate, linking it to cancer development (Lee et al., 2021; Meitzler et al., 2019; Valera et al., 2009). Other studies associate it with increased free radical levels, oxidative stress, and inflammation (Vilema-Enríquez et al., 2016). Yet, no clear consensus exists. The concentration of sodium perborate is often emphasized, underscoring the importance of early safety and efficacy testing (Sardaro et al., 2019). Regulatory agencies such as Brazilian National Health Surveillance Agency (ANVISA) have established concentration limits for sodium perborate in oral care products at 0.1% and other products at 3.0%, based on scientific evidence. Exceeding these limits may result in cytotoxic effects, particularly through increased oxidative stress and DNA damage (ANVISA, 2022). Our study ensures that the sodium perborate concentrations tested (0.06 mg/mL) remain well within these limits, supporting its safety in commercial formulations.

Literature controversies stem from the varied sodium perborate concentrations used in tests, complicating comparability, and repeatability. Studies frequently use high doses, not aligning with typical product concentrations (around 0.1 mg/mL), leading to *in vitro* effects that may not apply to human use (Attin et al., 2004).

The selection of the SkinEthic™ reconstructed human epidermis (RHE) skin model in this study was primarily due to the unavailability of validated 3D oral mucosal models in Brazil. Despite the existence of oral epithelium models such as SkinEthic™ HOE/Human Oral Epithelium (EpiSkin), EpiOral, and EpiGingival (MatTek Corp.), the complex bureaucratic process involved in importing these models makes it impractical for them to be used in timely testing within Brazilian laboratories. In contrast, the SkinEthic™ RHE model is widely accepted by regulatory bodies such as the OECD for irritation and toxicity evaluations, known for its reliability and reproducibility in toxicological testing. Nevertheless, it is important to interpret results from the skin model with caution when applying them to oral tissues, due to physiological differences between skin and mucosa, which may result in an underestimation of toxicity for substances intended for oral application. While the SkinEthic™ RHE model provides valuable preliminary data, future studies should focus on oral-specific mucosal models to confirm the findings. The results from this skin model offer a foundational understanding that can be complemented by oral epithelium models to further investigate the safety of sodium perborate in oral care applications.

Most studies evaluating sodium perborate use a 2D cell model, showing higher sensitivity to drug exposures than 3D culture models (Imamura et al., 2015; Muguruma et al., 2020). This underscores the importance of employing standardized culture models *in vitro* to minimize variability and ensure the results are transferable for regulating and producing safe oral care products (Duval et al., 2017). 2D models, though valuable for preliminary screenings, often exhibit higher sensitivity to drug exposures, which may not fully translate to *in vivo* conditions. On the other hand, 3D models provide a more physiologically relevant environment by mimicking the complex cellular interactions found in human tissues. This combination of 2D and 3D models in our study ensures that the findings are robust and transferable to human contexts, enhancing the reliability of toxicity predictions.

Considering the extensive use of sodium perborate in dentistry for applications such as biofilm reduction, gingivitis, periodontitis, and post-surgical treatments, it is essential to ensure the safety of oral care products containing this compound (Urban et al., 2019; Akuji and Chambers, 2017). With ongoing debates regarding its optimal concentration and potential effects, thorough exploration of its dose-response relationship and toxicity through various *in vitro* models remains critical for establishing safe usage guidelines (Duval et al., 2017; Urban et al., 2019; Akuji and Chambers, 2017; İpek et al., 2023).

This study assessed the cytotoxic effects of pure sodium perborate and oral care products containing it, using various cell models—monolayer (2D), spheroids (3D), and RHE. These models were chosen to increase the relevance and reliability of the findings for *in vivo* conditions. Primary gingival fibroblasts and human epidermal keratinocytes were employed, closely mimicking oral mucosa, to assess cell viability and proliferation. The products tested are commercially available for both professionals and the public, and key tests adhered to OECD Test Guideline 439. The outcomes of this study are intended to inform safer product formulations and use in the oral care industry, highlighting potential health risks associated with cytotoxicity.

## 2 Materials and methods

This study assessed the cytotoxicity of sodium perborate (BNaO_3_ · 4H_2_O–Sigma-Aldrich–CAS: 10486-00-7, Pro Analysis—p. a. 96%), a primary ingredient in oral care products, at a concentration of 0.06 mg/mL, higher than the highest concentration found in these products (around 0.015 mg/mL) and various other concentrations (0.001, 0.01, 0.1, and 1 mg/mL) prepared through serial dilution. Sodium perborate was dissolved in Dulbecco’s Modified Eagle Medium (DMEM) with both high and low glucose, tailored to the specific culture medium requirements of each cell type (DMEM high glucose for keratinocytes and DMEM low glucose for fibroblasts), to prepare serial dilutions for the cytotoxicity assays. The preparation followed the OECD Guide No. 129, starting with concentrations of 200 mg/mL and 20 mg/mL, with the higher concentration serving as the stock solution for subsequent dilutions.

Due to precipitation observed at the 200 mg/mL concentration, the 20 mg/mL concentration was selected to ensure complete solubility, preventing potential interference with the cytotoxicity assays. To prepare the 20 mg/mL sodium perborate solution, the required amount was precisely weighed and dissolved in the appropriate volume of DMEM (high or low glucose, depending on the cell type). Serial dilutions were then performed from this solution for the subsequent tests, including preparation of the 0.06 mg/mL solution.

The Bluem® commercial products are available, tailored for specific oral health conditions, include Oral Fluid, Oral Foam and Oral Mouthwash. Detailed information regarding their composition, use, sodium perborate concentration, and exposure time is provided in Supplementary Figure 1. Cytotoxicity was assessed using both two-dimensional (2D) and three-dimensional (3D) spheroid models. Given the short exposure times, dilution in culture medium was unnecessary, and the exposed volume per well was 100 µL.

To evaluate additional products, we used a reconstructed human epidermis model (SkinEthic™ RHE). Oral Gel, formulated to aid tissue healing, and Oral Cream, designed for the prevention and treatment of gingivitis, periodontitis, and peri-implantitis, were selected due to their specific viscosities and textures. Owing to their viscous nature, these formulations were tested exclusively in the reconstructed epidermis model, which provides an appropriate air-liquid interface. The spheroid and 2D models, typically optimized for liquid formulations, were deemed unsuitable for these products due to challenges in handling viscous substances.

All experiments included sodium dodecyl sulfate (SDS) at a concentration of 200 mg/mL as a positive control, solubilized in DMEM (high and low glucose) for the same exposure times as the other products and sodium perborate. SDS was chosen as a positive control due to its well-established cytotoxicity, providing a reliable benchmark for comparing the effects of sodium perborate and the commercial products. It is important to note that all tests adhered to the Good Laboratory Practice (GLP) standards.

### 2.1 Gingival fibroblasts and keratinocytes in culture monolayer (2D)

Primary human gingival fibroblasts (HGF–from Rio de Janeiro Cell Bank–Banco de Células do Rio de Janeiro–BCRJ Cod# 0089) and human keratinocytes (HaCat–BCRJ Cod# 0341) were cultured in Dulbecco’s modified Eagle medium: low glucose for HGF and high glucose for HaCat (DMEM—Gibco, Thermo Fischer Scientific, Massachusetts, United States). This medium was enhanced with 10% fetal bovine serum–FBS (Gibco, Thermo Fischer Scientific, Massachusetts, United States) and maintained at 37°C in a 5% CO_2_ humidified environment. Tests for bacteria, fungi, and *mycoplasma* were conducted to ensure sterility. For bacteria and fungi, the cell culture supernatant was introduced to thioglycolate (TIO) and Tryptic Soy Broth (TSB) (both from Acumedia), incubating for 14 days aerobically at 22.5°C ± 2.5°C and 32.5°C ± 2.5°C, respectively. *Mycoplasma* contamination in cell supernatants was identified via bioluminescence using the MycoAlert™ PLUS *Mycoplasma* Detection Kit (MycoAlert^®^, Lonza).

### 2.2 Gingival fibroblasts and keratinocytes in culture spheroids (3D)

To generate spheroids, U-bottom plates (Corning) were layered with a fine coat of sterile 1% high-purity agarose (Sigma-Aldrich Cas No 9012-36-6). Varied quantities of HGF and HaCat cells (1 × 10^4^, 2 × 10^4^, 3 × 10^4^, and 4 × 10^4^ cells/well) were cultivated for 3 days at 37°C in a 5% CO_2_ humidified environment until spheroids developed. The spheroids growth, form, and structure were inspected using an inverted light microscope (Nikon Eclipse). Images were snapped using ScopePhoto Leica software at a ×10 magnification (LAS EZ, Leica). For every experimental condition, the diameters of 10 spheroids were photographed and assessed with the ImageJ program (version 2015).

### 2.3 Skin irritation test

The skin irritation test was performed following OECD TG 439 (OECD, 2021). The study utilized the commercial SkinEthic™ RHE model as the reconstructed human epidermis (RhE). Maintenance followed the manufacturer’s guidelines. The RhE model is developed from normal human cells cultured at an air-liquid interface, forming the *stratum corneum* and keratin layer (De Vecchi et al., 2018; Kandárová et al., 2006). This model boasts organized basal, spiny, and granular layers and a multilayered *stratum corneum*. The intercellular lamellar lipid layers within this structure mirror major lipid classes found *in vivo*.

### 2.4 Cytotoxicity analysis

#### 2.4.1 Cell viability assay in culture monolayer (2D)

To evaluate cell viability in the 2D monolayer, HGF cells were seeded in 96-well plate at 2 × 10^4^ cells/well density and HaCat at 5 × 10^4^ cells/well, targeting 70% cell confluence. Once 70% confluence was attained, the cells were treated with sodium perborate (0.06 mg/mL) and various commercial oral care products for 30–60 s, in line with manufacturer-recommended exposure times (as detailed in Supplementary Figure 1 for each product).

In clinical or domestic applications, such as the use of oral hygiene products, exposure to the oral mucosa often occurs with products in their pure or highly concentrated form, particularly over short periods of time.

For alternative sodium perborate concentrations (0.001, 0.01, 0.1, and 1 mg/mL), a 10 min exposure was employed with the aim of extrapolating the recommended time and evaluating possible differences in cellular behavior in longer exposure.

Post-exposure, cells were rinsed with PBS and then treated with 100 µL of a basal medium infused with 1 mg/mL MTT (3-4,5-dimethylthiazole bromide-2-yl-2,5-diphenyltetrazolium - Sigma-Aldrich CAS, 298-93-1). After a 3 h incubation, the supernatant was removed, and samples were mixed with 100 µL of dimethyl sulfoxide (DMSO). Absorbance readings were taken at 570 nm for fibroblasts and 590 nm for keratinocytes.

SDS at 200 mg/mL was the positive control for cytotoxicity after a 30–60 s exposure, consistent with the solubility guidelines in OECD Test Guideline No. 129 (Joint Meeting Of The Chemicals Committee And The Working Party On Chemicals, 2010).

#### 2.4.2 Real-time electrical impedance cell proliferation monitoring

Proliferation was evaluated using real-time electrical impedance monitoring in E-Plate View xCELLigence RTCA SP 96-well plates with gold microelectrodes (ACEA Biosciences, San Diego, CA, United States). These plates operate at an electrical potential of 22 mV. The cytotoxicity assay adhered to the manufacturer’s protocol. To set up the experiment, each well was filled with the complete basal medium and equilibrated at 37°C. Cells were then seeded, and the plate was positioned back into the xCELLigence station for real-time tracking, with data recorded hourly.

After a 24 h adhesion phase, cells were exposed to various sodium perborate concentrations and the specified commercial products for either 30–60 s or 10 min in separate tests. Monitoring continued for an additional 24 h, summing up to 48 h of total observation. After exposure, the cells were maintained in culture medium with 10% FBS until the end of the assay.

Impedance, or resistance offered by cells, is denoted as the Cell Index (CI). The formula used is CI = (Zi-Z0) [ohm]/15 [ohm], where Zi is resistance at a particular moment, and Z0 is the initial resistance. To obtain a normalized CI, the CI at a specific time (CIti) is divided by the CI when normalization began (CInml_time). The resulting data were automatically generated and processed using dedicated software linked to the monitoring device. The controls in this phase comprised a medium without cells, untreated cells, and SDS. The assays were conducted in accordance with established procedures (Veschi et al., 2024).

#### 2.4.3 Cell viability assay in culture spheroids (3D)

**FIGURE 1:**
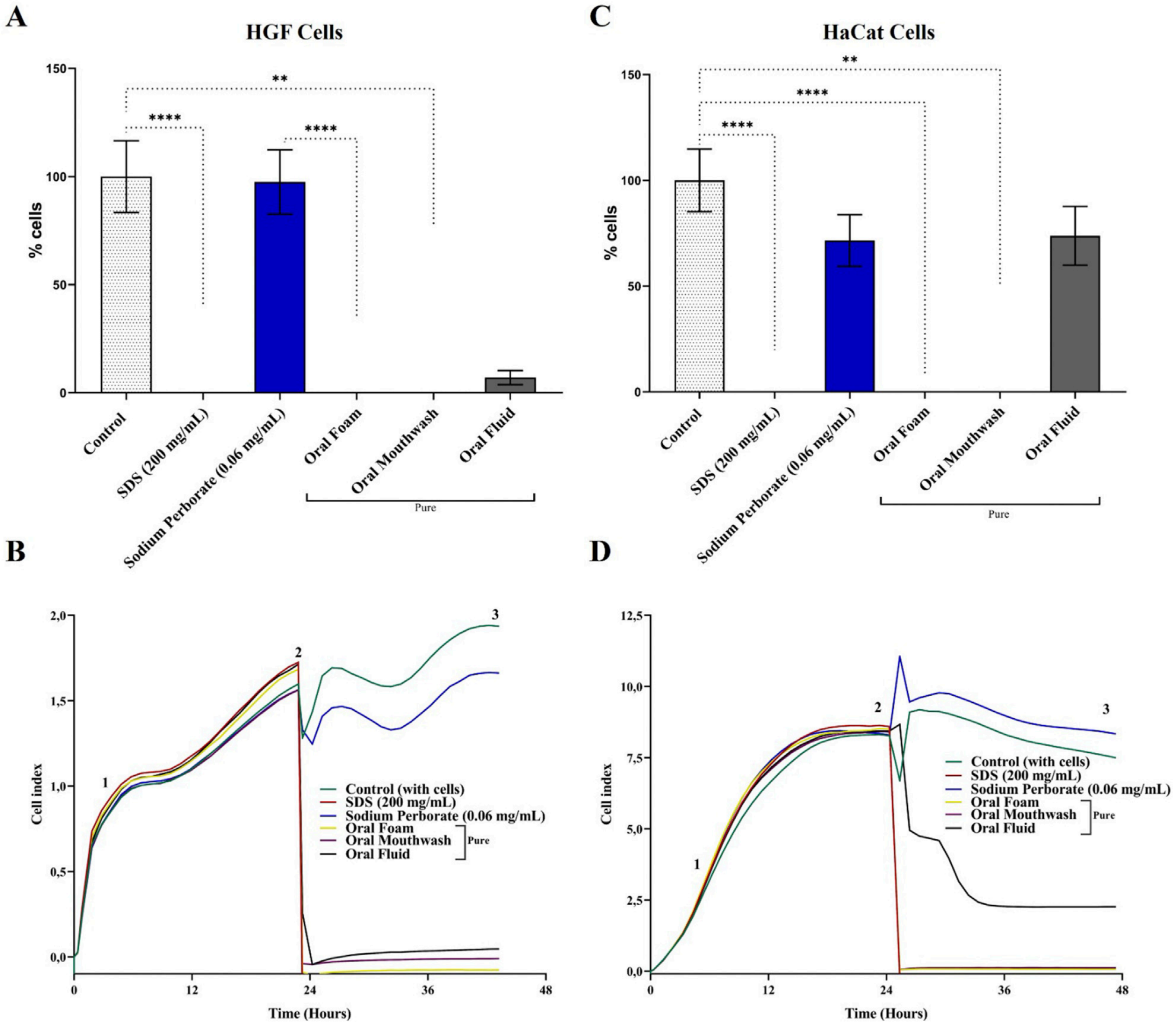
Viability and proliferation analysis of HGF and HaCat cells in 2D monolayer culture. **(A)** Viability of HGF cells was assessed using the MTT assay after a 30–60 s exposure to 0.06 mg/mL sodium perborate, 200 mg/mL SDS, and various oral care products: Oral Foam, Oral Mouthwash, and Oral Fluid. The bars represent the mean ± 95% confidence interval (n = 6). **(B)** Proliferation dynamics of HGF cells following a 30–60 s exposure to sodium perborate and oral care products (n = 6, triplicate), evaluated by electrical impedance (xCELLigence). **(C)** Viability of HaCat cells assessed using the MTT assay after 30–60 s exposure to 0.06 mg/mL sodium perborate, 200 mg/mL SDS, and various oral care products: Oral Foam, Oral Mouthwash, and Oral Fluid. The bars represent the mean ± 95% confidence interval (n = 6). **(D)** Proliferation dynamics of HaCat cells after 30–60 s exposure to sodium perborate and oral care products (n = 6, triplicate), assessed by electrical impedance (xCELLigence). The graph shows three phases: 1) Initial adhesion and proliferation without treatment, 2) Proliferation post-treatment, and 3) Plateau. The basal medium was used as a control, while SDS served as a positive control for cell death. Statistical significance is indicated as * (*p* < 0.05), ** (*p* < 0.01), *** (*p* < 0.001), **** (*p* < 0.0001).

To determine the ideal cell density for testing, the cell models were characterized at various densities, as shown in Complementary Figure 1. Based on these results, a cell density of 3 × 10⁴ cells/well in 96-well U-bottom plates coated with 1% agarose was selected for HGF and HaCat spheroids, considering their average size. Three days after spheroid formation, the cells were treated with the specified concentrations of sodium perborate and oral care products. Cell viability was assessed using flow cytometry, with spheroids dissociated prior to analysis. Approximately 2.4 × 10⁵ cells/mL (averaging eight spheroids per condition) were stained using the FITC Annexin V Apoptosis Detection Kit (Biolegend, 640914), following the manufacturer’s instructions. Cells were washed three times with phosphate-buffered saline (PBS, 0.01 M), followed by incubation with 0.125% trypsin (Sigma-Aldrich) in a humidified 5% CO₂ incubator at 37°C for 5 min. Trypsin activity was halted by adding culture medium containing 10% FBS, and cells were mechanically dissociated. The cells were then centrifuged at 500 g for 7 min at 4°C. The resulting pellet was resuspended in 100 μL of annexin-binding buffer and incubated at room temperature for 15 min with 5 μL of annexin/fluorescent binding solution (FITC) and 10 μL of propidium iodide, as per the manufacturer’s instructions. All analyses were conducted using a FACS Aria III flow cytometer (BD Biosciences), with a recommended event count of 20,000 per analysis, and the data were interpreted using FlowJo software.

Unexposed cultured cells in basal medium served as the negative control, while SDS was used as the positive control. The structure and appearance of the spheroids were continuously monitored using an inverted light microscope (Nikon Eclipse), and images were captured with ScopePhoto Leica software. Spheroid diameters were measured as described previously.

#### 2.4.4 Cell viability assay in reconstructed epidermis model

The SkinEthic™ RHE reconstructed epidermis model, commercially sourced from EpiSkin™, was used to assess cytotoxicity following exposure to pure sodium perborate and oral care products, including Oral Gel and Oral Cream. These products were not tested in previously mentioned models due to their specific constraints and the need for dilution, which would have altered the original sodium perborate concentration. Although the SkinEthic™ RHE model is skin-like, it was selected post-formation because it histologically resembles mucosa. The cytotoxicity assessment adhered to OECD TG 439, which outlines the *in vitro* skin irritation test for reconstructed human epidermis. The commercial products and sodium perborate were tested at a concentration of 0.06 mg/mL, with SDS serving as a positive control. Following a 17-day formation period of the reconstructed epidermis, the test substances were applied for 42 min. This was followed by a 42-h recovery period, after which cell viability was assessed using the MTT assay at 570 nm, as previously described.

### 2.5 Statistical analysis

Descriptive statistical analysis was employed on the collected data. Upon confirming data normality using the Shapiro-Wilko test, subsequent analyses were conducted using the ANOVA and Tukey’s test, with a significance level set at 0.05.

## 3 Results

### 3.1 Cell viability and proliferation assay in culture monolayer (2D)

HGF and HaCat cells in a 2D monolayer were subjected to a 0.06 mg/mL concentration of sodium perborate. Additionally, these cells were exposed to Oral Foam, Oral Mouthwash, and Oral Fluid for 30–60 s, according to the manufacturer’s guidelines outlined in Supplementary Figure 1. Subsequently, MTT and electrical impedance methods assessed cell viability and proliferation.

HGF cells exhibited fibroblastoid characteristics, as depicted in Supplementary Figure 2A. Notably, a decline in HGF viability was observed after exposure to oral care products. However, the viability was comparable to the control group (cells in basal medium), showing no statistically significant reduction when exposed to the 0.06 mg/mL concentration of sodium perborate (Figure 1A). Real-time electrical impedance measurements further validated the cytotoxic effects of the oral care products, with SDS serving as a benchmark for cytotoxicity (Figure 1B).

The effects of sodium peroxide at varying concentrations (1, 0.1, 0.001, 0.001 mg/mL) on HGF cells were examined over a 10 min exposure, which exceeds the manufacturer’s recommended duration. A noticeable reduction in viability (Supplementary Figure 3A) and proliferation (Supplementary Figure 3B) was only observed at the 1 mg/mL concentration compared to the control.

HaCat cells exhibited an epithelial morphology, as illustrated in Supplementary Figure 2B. When assessing viability, consistent findings emerged. Exposure to a 0.06 mg/mL concentration of sodium perborate showed no significant impact on cell viability compared to the control group (cells maintained in basal medium). However, when evaluating with both the MTT assay and real-time electrical impedance, a decline in cell viability was noted upon exposure to oral care products (Figures 1C, D, respectively). When the exposure duration was extended to 10 min, only the higher 1 mg/mL concentration led to a significant reduction in viability and proliferation, as depicted in Supplementary Figures 4A, B, respectively.

### 3.2 Optimization of HGF and HaCat spheroids

To refine the three-dimensional (3D) model, various cell densities (1 × 10^4^, 2 × 10^4^, 3 × 10^4^, 4 × 10^4^ cells/well) of both HGF and HaCat were seeded in 96-well round-bottom plates. These plates were pre-coated with a 1% agarose layer to prevent cell adhesion. Spheroids were successfully formed after a 3-day culture period, as depicted in Supplementary Figure 5A schematic representation. The spheroids retained their rounded morphology irrespective of the cell density used (Supplementary Figure 5B). Predictably, the diameter of the spheroids grew proportionally with the initial cell count, a trend evident in both cell models as illustrated in Supplementary Figure 5C. Spheroids cultivated with a density of 3 × 10^4^ cells/well achieved an average diameter of approximately 410 μm, making them ideal candidates for subsequent experiments. The choice of a density of 3 × 10^4^ cells/well was based on optimal parameters for cell viability and spheroid size.

### 3.3 HGF viability assay in culture spheroids

**FIGURE 2:**
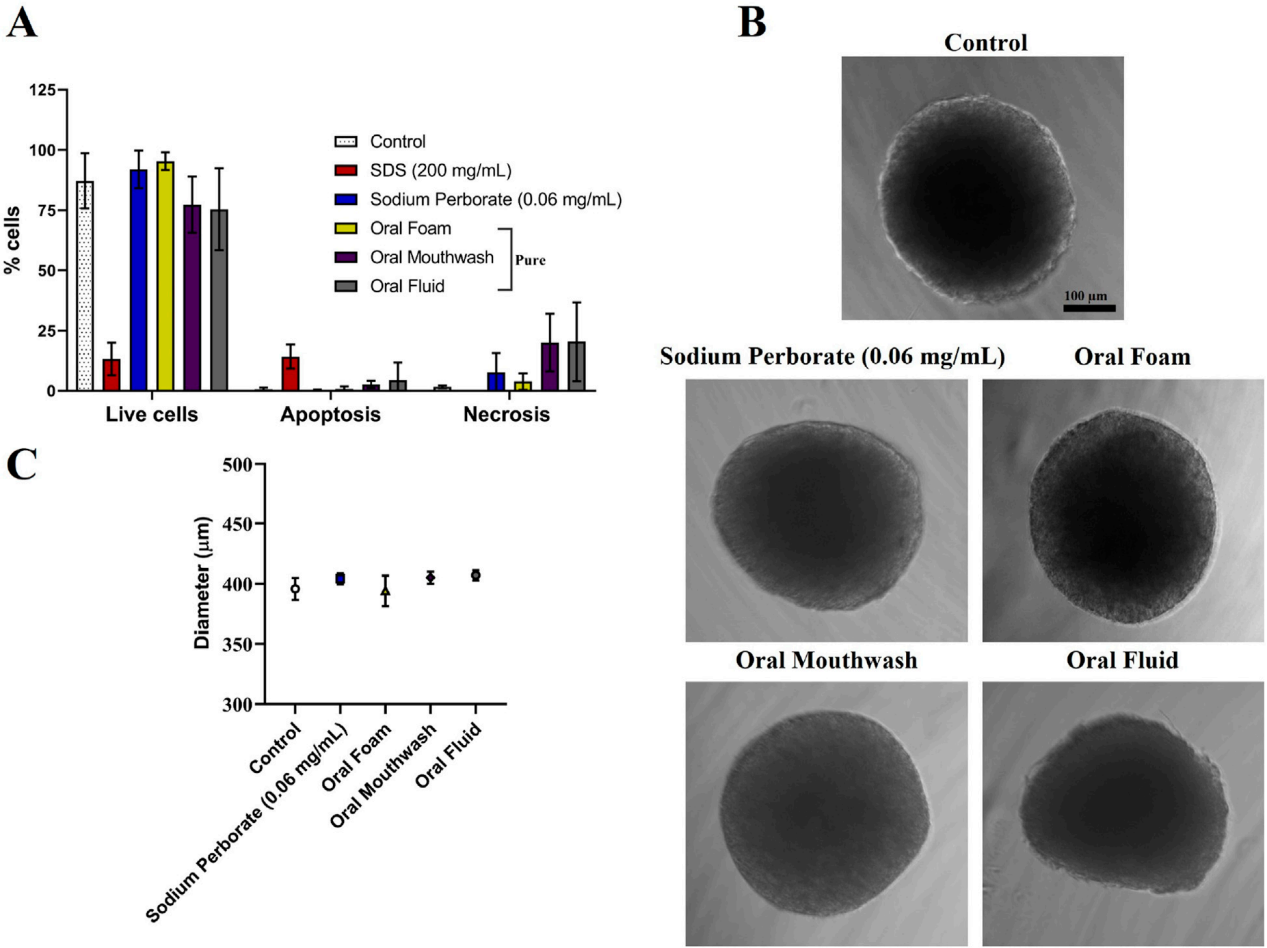
Analysis of HGF spheroids after exposure to sodium perborate and various oral care products. **(A)** Flow cytometry analysis of spheroid viability (live and dead) following exposure to 0.06 mg/mL sodium perborate, 200 mg/mL SDS, and oral care products: Oral Foam, Oral Mouthwash, and Oral Fluid. **(B)** Phase contrast images showing the morphology of treated HGF spheroids (scale bar: 100 µm). **(C)** Graph illustrating the average diameter (µm) of HGF spheroids after treatment. The results in the graphs are presented as mean values with a 95% confidence interval. All assays were performed in triplicate.

HGF spheroids were subjected to a 30–60 s exposure to sodium perborate (0.06 mg/mL) and various oral care products, including Oral Foam, Oral Mouthwash, and Oral Fluid. Viability post-exposure was assessed using the live and dead flow cytometry assay. The viability of HGF spheroids remained consistent post-exposure to sodium perborate and oral care products, with no significant changes in cell viability observed, indicating a lack of cytotoxic effects at these exposure conditions (Figure 2A). Furthermore, no noticeable changes were observed in the morphology (Figure 2B) or the average diameter (Figure 2C) of the spheroids. When spheroids were exposed for an extended duration (10 min) to varying concentrations of sodium perborate (1, 0.1, 0.001, 0.001 mg/mL), neither the viability (Supplementary Figure 6A) nor the morphology and size (Supplementary Figures 6B, C) were significantly affected.

### 3.4 HaCat viability assay in culture spheroids

**FIGURE 3:**
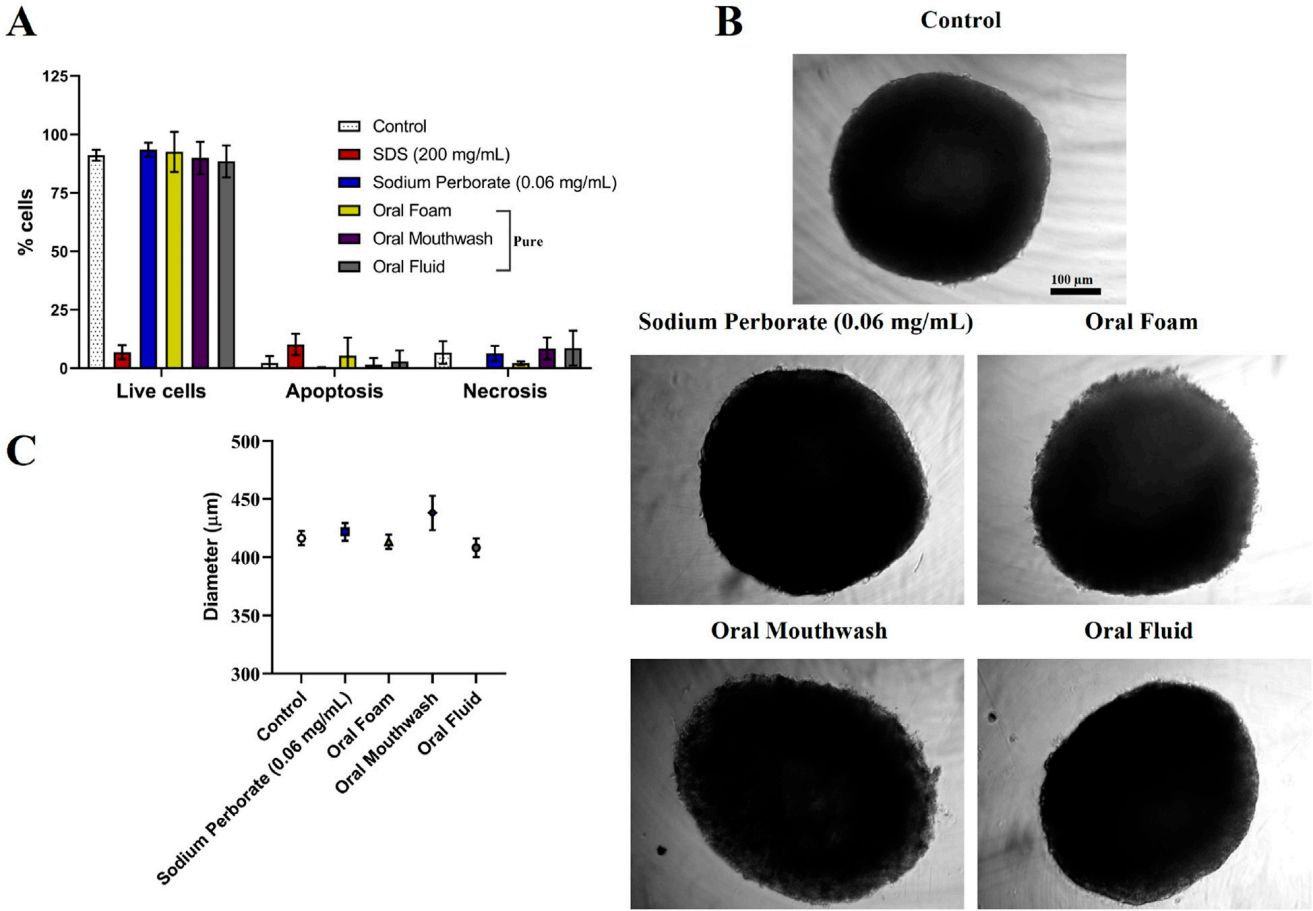
Assessment of HaCat spheroids after exposure to sodium perborate and various oral care products. **(A)** Flow cytometry results depicting spheroid viability based on the live-dead assay after treatment with 0.06 mg/mL sodium perborate, 200 mg/mL SDS, and oral care products: Oral Foam, Oral Mouthwash, and Oral Fluid. **(B)** Phase contrast images showing the morphology of treated HaCat spheroids (scale bar: 100 µm). **(C)** Graph representing the average diameter (µm) of treated HaCat spheroids. The results in the graphs are presented as mean values with a 95% confidence interval. All assays were performed in triplicate.

HaCat spheroids displayed comparable outcomes as HGF. Viability remained consistent after exposure to sodium perborate (0.06 mg/mL), and oral care products, as shown in Figure 3A (at 30–60 s exposure) and various concentrations of sodium perborate (0.001, 0.001, 0.1, 1 mg/mL) as shown in Supplementary Figures 7A–C (at 10 min exposure). No significant alterations in spheroid morphology or average diameter were observed, as evidenced in Figures 3B, C.

### 3.5 Cell viability assay in reconstructed epidermis model

**FIGURE 4:**
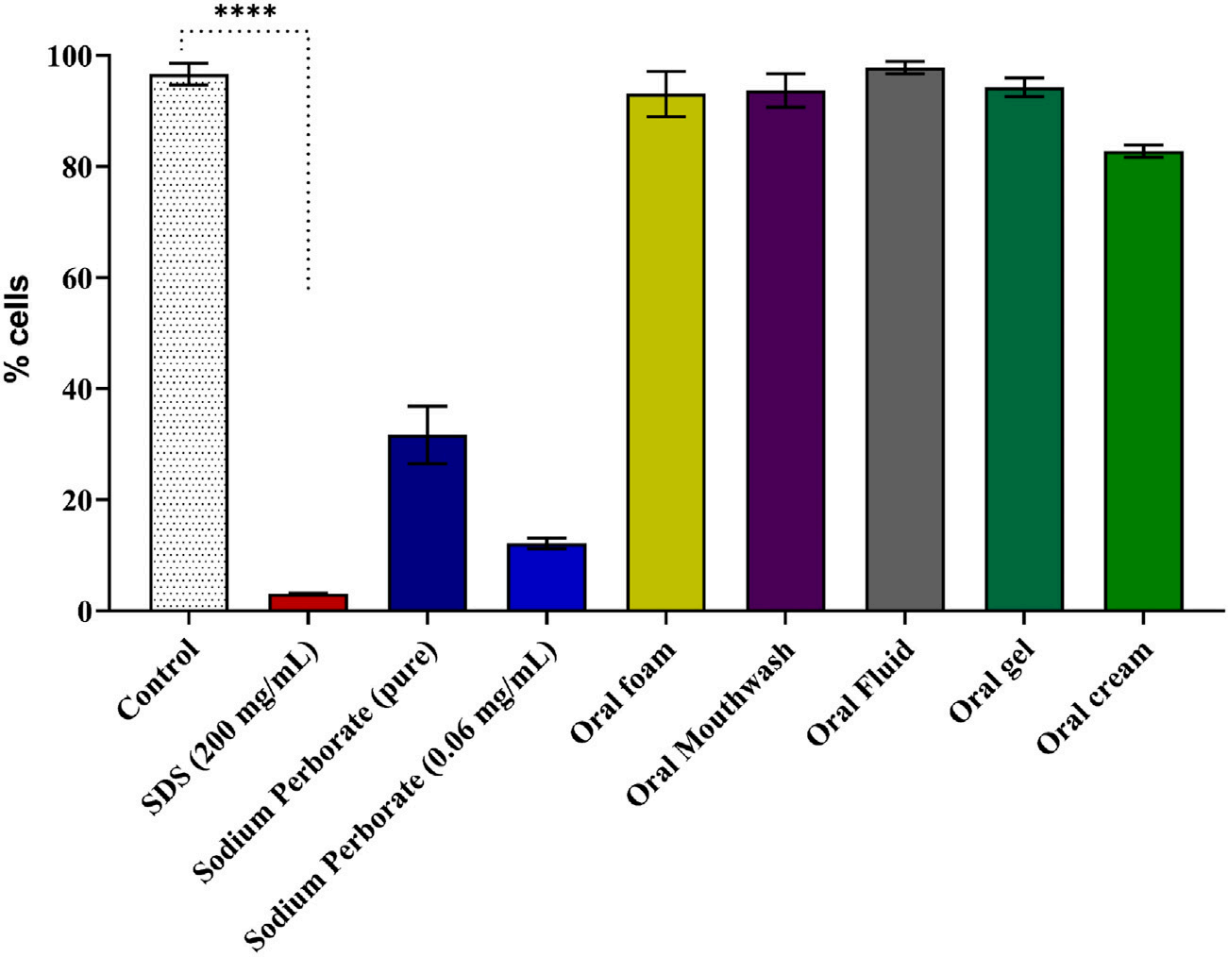
Viability assessment of reconstructed epidermis using the MTT assay. Results of the MTT assay following a 42-minute exposure to sodium perborate (pure and at 0.06 mg/mL) and oral care products: Oral Foam, Oral Mouthwash, Oral Fluid, Oral Gel, and Oral Cream. The basal medium served as the control, with SDS as the positive control for cell death. Statistical significance is denoted as **** (*p* < 0.0001). The bars represent the mean ± 95% confidence interval (n = 3).

The experiment followed OECD TG 439 which describes the conduct of *in vitro* skin irritation using a reconstructed human epidermis model. After a 17-day formation period, resembling mucosal histology (Supplementary Figure 8), the model was exposed to sodium perborate and various oral care products, including Oral Gel and Oral Cream, which were incorporated at this stage due to their high viscosity. The exposure lasted for 42 min, followed by a 42-h recovery period, after which cell viability was assessed using the MTT assay. The results revealed that pure sodium perborate and its 0.06 mg/mL concentration significantly reduced cell viability compared to the control group cultured in a basal medium. Despite the prolonged 42 min exposure, none of the tested commercial oral care products resulted in decreased cell viability, underscoring their safety profile under the tested conditions in Figure 4.

## 4 Discussion

Sodium perborate releases hydrogen peroxide and nascent oxygen when it comes into contact with water (Mishra et al., 2020). This property has found its application in dental products for tooth whitening and post-treatment, utilizing the antiseptic and oxidizing characteristics of the produced compounds (Alkahtani et al., 2020). Beyond dental care, hydrogen peroxide is traditionally employed to cleanse minor wounds and to aid in the removal of foreign objects from them (Alkahtani et al., 2020; Murphy and Friedman, 2019).

However, there are concerns with sodium perborate’s potential cytotoxic effects. Some *in vitro* studies have shown a dose-dependent decrease in epithelial cell viability upon exposure (Zhang et al., 2017). At heightened concentrations, sodium perborate can produce reactive oxygen species (ROS), leading to DNA damage, genotoxicity, and cytotoxicity (Marto et al., 2020). Sodium perborate’s efficacy in dental applications is well-documented. Studies have confirmed its ability to promote tooth whitening and eliminate biofilms without compromising cell proliferation and viability (Marto et al., 2020; Malkondu et al., 2011; Jessop et al., 2021). This compound’s action largely hinges on generating reactive oxygen species (ROS). However, these ROS, while potent, often face challenges in penetrating the cell membrane or traveling extensive intracellular distances (Lin et al., 2019).

Antioxidants play a pivotal role in counteracting the potential harm posed by ROS. When ROS levels eclipse the cellular protective capacity, they can inflict health damage (Jessop et al., 2021; Iglesias-Pedraz and Comai, 2020). Catalase is instrumental in neutralizing hydrogen peroxide, and any reduction in its activity can lead to an accumulation of this compound, triggering a cascade of potentially deleterious effects (He et al., 2017). The body’s defense against such scenarios involves antioxidants. These molecules work in tandem with enzymes like superoxide dismutase (SOD), glutathione peroxidase, and catalase to curtail the cellular damage instigated by ROS (Naik et al., 2006; Yamada et al., 2012).

ROS, particularly hydrogen peroxide, have been implicated in cellular signaling pathways (Di Marzo et al., 2018). They can function as secondary messengers and are known to enhance immune responses. Such interactions can bolster the wound-healing process post-injury (Atrux-Tallau et al., 2011).

Within dentistry, one of the emerging concerns is the potential of these compounds to cause root resorption. Root resorption is characterized by the gradual loss of the mineralized tissue that composes the tooth structure (Hossainian et al., 2011). Interestingly, the severity of this phenomenon appears to be contingent on the concentration of sodium perborate employed. Studies indicate that concentrations around 35% can result in intense reduction in viability, while concentrations near 8% seem benign (Soares et al., 2015). This dichotomy underscores the importance of concentration and exposure time in determining the effect of sodium perborate. While much of the literature focuses on the observations from clinical trials or investigations using, extracted teeth, (Atrux-Tallau et al., 2011; Soares et al., 2015), there is a noticeable paucity of *in vitro* studies on cell models (Lin et al., 2019; Zhang et al., 2017).

The current work employed a range of cell culture models to address these gaps, encompassing monolayer (2D) and three-dimensional (3D) configurations (Duval et al., 2017). The rationale behind the choice of these models was twofold. While 2D models offer cost-effective preliminary testing, 3D models provide more robust and physiologically relevant data with their more intricate cellular structures and interactions (Cacciamali et al., 2022). The 2D monolayer model remains useful for preliminary screenings due to its cost-effectiveness, but its limited cell-cell and cell-matrix interactions reduce its predictive value for complex tissue environments (Jensen and Teng, 2020). In contrast, 3D models, such as spheroids, better simulate the *in vivo* environment, leading to more physiologically relevant results, as they allow for enhanced cellular interactions and more accurate toxicity assessments (Achilli et al., 2012).

Research on monolayer macrophages exposed to varying concentrations of sodium perborate has provided insights into its biocompatibility (Asfora et al., 2005). The study by Asfora et al. (2005), which focused on assessing the biocompatibility of sodium perborate for tooth whitening applications, observed no significant morphological changes in the macrophages post-exposure. In a separate investigation involving fibroblasts, the combined effects of sodium perborate and tetraacetylethylenediamine (an activator in bleaching procedures) were examined, and results underscored the dose-dependent toxicity of the combination (Simbula et al., 2010).

Our current study observed that both fibroblasts and keratinocytes experienced significant cell death at elevated concentrations (1 mg/mL and above), while no effects were noted at lower concentrations (0.0025%). These findings were further supported by MTT assay and electrical impedance tests, revealing viability reductions at concentrations of 0.1 and 1 mg/mL. However, concerns about its safety have arisen in studies, prompting uncertainty in the industry and among regulators about product registration, necessitating further tests and safety evaluations.

In our study, the 2D HaCat cell model was exposed to various commercial products for intervals of 30–60 s and 10 min. Cytotoxic effects became evident in these conditions, as evidenced by loose cells in the supernatant and notably reduced viability in the MTT and electrical impedance assays. Nevertheless, these cytotoxic effects were absent in the spheroid and reconstructed epidermis models. This divergence hints at potential limitations of the 2D monolayer model, especially concerning diminished cellular and extracellular matrix interactions. Similarly, the gingival fibroblast model (HGF) revealed reduced viability in the 2D model upon product exposure. Contrastingly, in the three-dimensional models, these products failed to induce any adverse effects within the manufacturer-recommended exposure time of 60 s.

We observed consistent viability regarding sodium perborate at shorter exposure durations (60 s), especially at concentrations mirroring those in commercial products (0.01 mg/mL and 0.06 mg/mL). The contrasting results between the 2D monolayer models and the reconstructed epidermis model may stem from the heightened sensitivity of the 2D model or the specific exposure times in the 3D models (Jensen and Teng, 2020; Gargotti et al., 2018). This sensitivity might be attributed to the limited cell-cell and cell-matrix interactions in the 2D model. The 3D models, in contrast, facilitate enhanced interactions, offering cells better protection against external exposures (Jensen and Teng, 2020).

Cell cultures fundamentally serve as proxies to approximate responses in living organisms. While the 2D monolayer model remains prevalent due to its cost-effectiveness, its inherent limitations render it less representative in isolation (Lelièvre et al., 2017). Consequently, 3D cultures have garnered attention for their potential to emulate living organisms, finding applications in diverse scientific domains, including toxicology (Cacciamali et al., 2022).

In the tests with oral care products, different effects were observed in the monolayer and three-dimensional models. In the HGF and monolayer HaCat model, MTT and electrical impedance results showed that these products could cause cell death. This was observed even at the manufacturer’s recommended 30–60-s exposures. The compositional complexity of the products should be taken into account. The oral foam, mouthwash, and oral cream contain multiple ingredients that may exhibit synergistic interactions, potentially mitigating the toxicity of sodium perborate.

Moreover, the absence of SDS, a recognized skin irritant, in the oral fluid may contribute to its reduced cytotoxicity. The oral cream, which contains sodium sulfate but lacks SDS, may similarly demonstrate a lower level of toxicity. Our findings did not identify sodium perborate as a contributing factor to toxicity at short exposure times. Therefore, the complexity of the product components may be a key determinant in the observed results. When comparing the spheroid (3D) model to the 2D monolayer, cell viability remained consistent during exposures of 30–60 s, paralleling findings in the negative control. Spheroids, by design, have enhanced cell-cell interactions and a dense cellular matrix, potentially limited product diffusion and reducing toxicity. This protective effect is especially evident at concentrations of 0.06 mg/mL, further suggesting that sodium perborate’s cytotoxicity might not be the sole concern.

For the reconstructed epidermis model, the OECD TG 439 recommends a prolonged exposure of 42 min. In this context, sodium perborate at 0.06 mg/mL reduced cell viability, whereas the tested oral care products did not. Two possible explanations arise: 1) the extended exposure time, exceeding manufacturer recommendations, may enhance the effects, potentially due to ROS generation by sodium perborate; and 2) sodium perborate’s lower viscosity compared to oral care products might allow more effective interaction with the reconstructed epidermis, influencing viability over longer periods.

The SkinEthic™ RHE model was selected due to the lack of validated 3D oral mucosal models in Brazil and its recognition by regulatory bodies like the OECD. Despite this, its limitations in replicating oral tissue toxicity should be considered, especially in comparison with available but less accessible models like EpiOral and EpiGingival. However, while this skin model provides valuable data, it may underestimate the toxicity of substances intended for oral application. Therefore, our results using the SkinEthic model should be interpreted with caution when extrapolating to oral tissues, given the physiological differences between skin and mucosal models.

From a regulatory standpoint, the ANVISA issued Resolution RDC No. 530 in August 2021. This regulation, based on solid scientific evidence, sets concentration limits for sodium perborate in oral care products (0.1%) and other products (3.0%) (ANVISA, 2022). For hydrogen peroxide, the limit is defined as 0.1% of H_2_O_2_, whether present or released. Importantly, the oral care products assessed in this study contain approximately 0.015% sodium perborate, which is well within these regulatory limits, reinforcing their safety. As regulatory limits set by ANVISA provide a framework for product safety. However, further research into the interactions between sodium perborate and other ingredients in oral care products could help refine these regulations, ensuring that they remain based on the most current and comprehensive scientific evidence.

Most clinical research on oral care products such as Blue®m mouth rinses and gels, focuses on patient outcomes following dental procedures. While *in vitro* studies are limited, clinical trials and observational studies have consistently demonstrated the efficacy of these products in addressing common oral health concerns, including biofilm-related bacterial infections (Cunha et al., 2019) oral mucositis (Di Stefano et al., 2022) post-surgery wound healing (Stroparo et al., 2021) periodontitis (Niveda and Kaarthikeyan, 2020). These products have been shown to accelerate tissue repair and reduce biofilm formation.

Both monolayer and three-dimensional models have their unique limitations. Ideally, comprehensive evaluations across models yield robust results. Given the unavailability of an equivalent mucosal model in Brazil, we opted for the reconstructed epidermis model, representative of the skin. Future endeavors should compare outcomes across equivalent mucosal models for enhanced insights.

**FIGURE 5:**
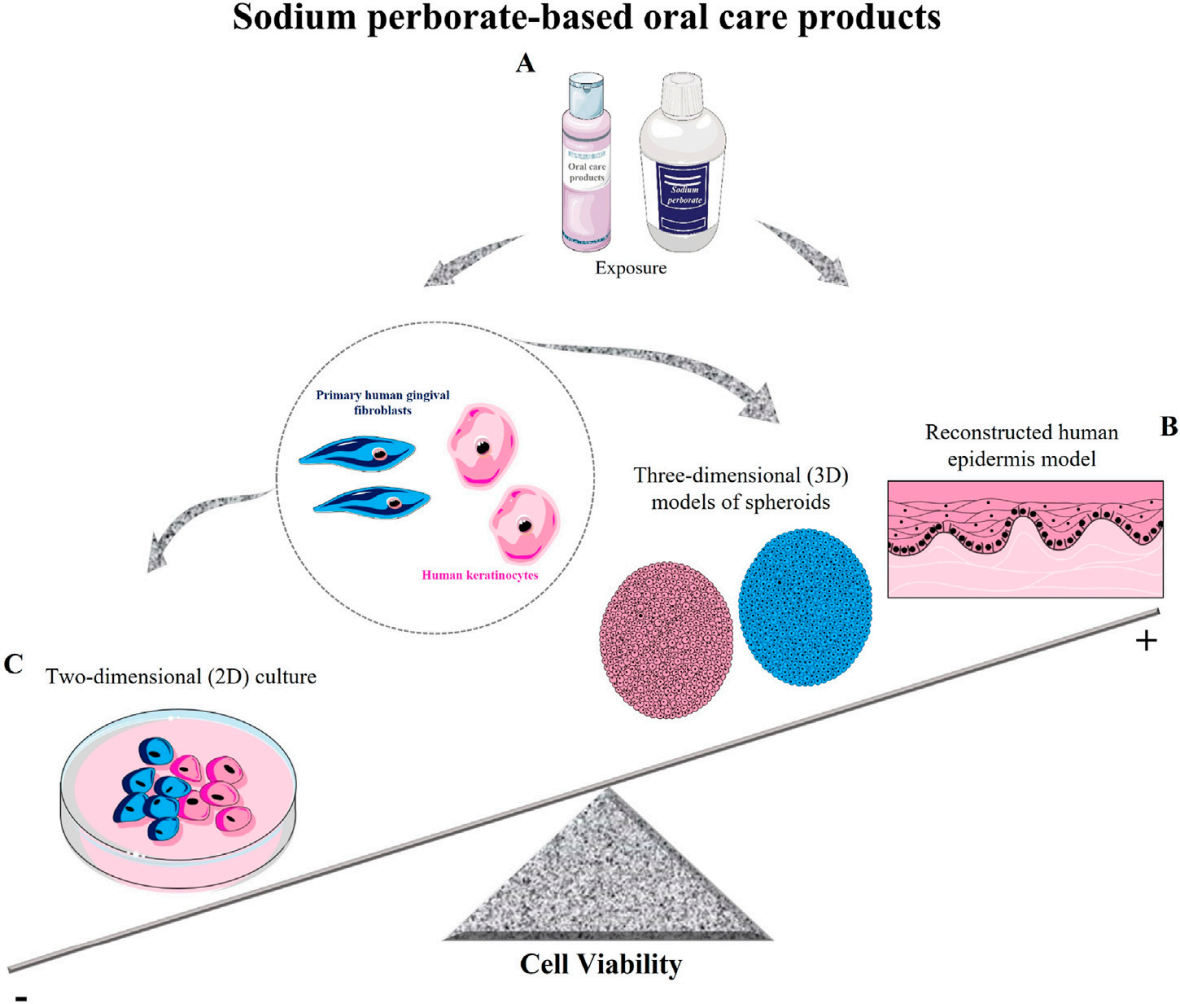
Schematic model of sodium perborate and sodium perborate-based oral care products on cell viability. **(A)** Sodium perborate and sodium perborate-based products were tested on primary human gingival fibroblasts and human keratinocytes in both monolayer (2D) and three-dimensional models (spheroids and reconstructed epidermis). **(B)** Sodium perborate-based products showed no significant toxicity in 3D models (spheroids and reconstructed epidermis). **(C)** In 2D monolayer cultures, a reduction in cell viability was observed. These results demonstrate the critical role of exposure time and model type (monolayer or 3D) in determining *in vitro* toxicity.

Methodological nuances introduce limitations, be it the chosen biological model or analytical metrics. Methodological differences, including the choice of biological models and analytical techniques, can significantly impact toxicity outcomes and the reproducibility of results. Future studies should aim to standardize protocols across different models to ensure more consistent and comparable findings. Additionally, the scientific literature occasionally presents ambiguities or scant details, potentially leading to reproducibility challenges. As for sodium perborate, despite some disagreements among researchers most publications, including our findings, converge on the idea that specific concentrations and exposure times dictate cytotoxic outcomes (Aschheim, 2015) (as depicted in Figures 5A–C). This highlights the importance of rigorous toxicity tests before broad-scale deployment, ensuring the delivery of safe, reliable products to consumers.

## 5 Conclusion

Our findings underscore the pivotal roles that exposure duration and chosen cell culture model play in *in vitro* toxicity determination. Specifically, while the tested products exhibited toxicity in monolayer cultures, they appeared non-toxic in three-dimensional (3D) models. Sodium perborate, in concentrations below 0.1 mg/mL, demonstrated minimal toxicity across both models. Furthermore, when evaluated using the reconstituted epidermis in alignment with the OECD TG 439 criteria, the products were determined to be non-irritating to the 17-day-old epidermis, partially mimicking an oral mucosa. However, concerns about its safety have arisen in studies, prompting uncertainty in the industry and among regulators about product registration, necessitating further tests and safety evaluations.

## Data Availability

The original contributions presented in the study are included in the article/Supplementary Material, further inquiries can be directed to the corresponding author.
