# Supplementary material for: A multimodal in vitro approach to assess the safety of oral care products using 2D and 3D cellular models
Source: Front Toxicol. 2024 Nov 6;6:1474583. doi: 10.3389/ftox.2024.1474583 (PMC11576945; doi:10.3389/ftox.2024.1474583)
Supplement: Supplementary file 1 [file DataSheet1.pdf]

A multimodal *in vitro* approach to assess the safety of oral care products using 2D and 3D cellular models

Marceli L. da Silva[1,2†], Wanderson de Souza[1,2†],
Rodrigo De Vecchi[3], Amanda Lopes[4], Tatiana Deliberador[4] and
Jose M. Granjeiro[1,2,4*]

[1]Divisão de Metrologia em Biologia, Diretoria de Metrologia Científica, Industrial e Tecnologia, Instituto Nacional de Metrologia, Qualidade e Tecnologia (INMETRO), Duque de Caxias-RJ, Brazil,

[2]Programa de Pós-Graduação em Biotecnologia, Instituto Nacional de Metrologia, Qualidade e Tecnologia (INMETRO), Duque de Caxias-RJ, Brazil,

[3]Episkin, Rio de Janeiro-RJ, Brazil,

[4]Instituto Latino Americano de Pesquisa e Ensino Odontológico —ILAPEO, Curitiba-PR, Brazil

†These authors have contributed equally to this work

*CORRESPONDENCE

Jose M. Granjeiro, jmgranjeiro@inmetro.gov.br

Supplementary Figures

Supplementary Figure 1: Bluem® products: The composition, indication of use, the concentration of sodium perborate and exposure time.

Oral Products Information				
**Product**	**Composition**	**Indication of use**	**Sodium Perborate concentration**	**Recommended usage time**
Oral Fluid	aqua, glycerin, sodium citrate, citric acid, sodium perborate, cellulose gum, sodium methylparaben, sodium saccharin	Sensitive mucous membranes, Gingivitis, Periodontitis, Periimplantitis, Conservation of dental implants, Post-operative surgeries, Oral injuries, Relieves the discomfort of patients during Chemotherapeutic treatments, Relieves the discomfort of patients during the treatment of Radiotherapy	0.006 mg/mL	60 sec
Oral Foam	aqua, glycerin, mel, sodium lauryl sulfate, PVP, sodium citrate, cellulose gum, parfum, sodium perborate, sodium methylparaben, citric acid, methyl salicylate, xylitol, lactoferrin, magnesium sulfate, D-LLimonene, CT 42090, linalool	Oral cleaning routine and for orthodontic appliances	0.003 mg/mL	60 sec

Mouthwash	aqua, glycerin, mel, sodium lauryl sulfate, PVP, sodium citrate, cellulose gum, parfum, sodium perborate, sodium methylparaben, citric acid, methyl salicylate, xylitol, lactoferrin, magnesium sulfate, D-LLimonene, CT 42090, linalool	Assists in the healing process, Post-operative surgeries, prevention and treatment of Gingivitis, Periodontitis and Peri-implants	0.003 mg/mL	30 sec
Oral cream	glycerin, aqua, hydrated silica, mel, silica, PEG-32, aroma, cocamidopropyl, betaine, cellulose gum, magnesium sulfate, sodium citrate, sodium saccharin, xylitol, lactoferrin, methyl salicylate, sodium chloride, sodium perborate, sodium methylparaben, citric acid, sodium sulfate, limonene, CI 42090, linalool	Daily use, Antiseptic and anti-inflammatory effect, prevention and treatment of Gingivitis, Periodontitis and Peri-implants	0.003 mg/mL	> 2 min
Oral gel	aqua, alcohol, glycerin, silica, sodium saccharin, sodium perborate, citric acid, sodium gluconate, PEG-32, xanthan gum, lactoferrin	Periodontal pocket reduction, maintenance of gingival health, prevention and treatment of periodontitis and peri-implants, antimicrobial, Post-operative surgeries, treatment of post-surgical complications, Stimulates the oral healing process, Oral injuries	0.015 mg/mL	not specified by the manufacturer

Supplementary Figure 1: Composition, indication for use, concentration of sodium perborate, and exposure time. All descriptions are provided by the manufacturer of the products - Bluem®.

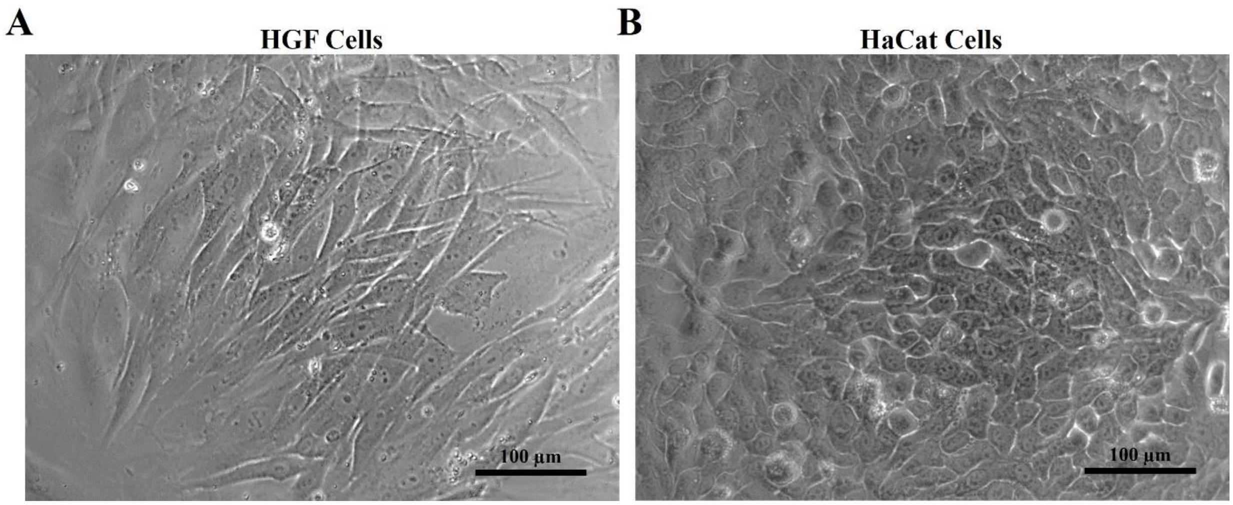

Supplementary Figure 2: Photomicrograph of HGF and HaCat cell morphology. Normal morphology of control HGF and HaCat cells is shown.

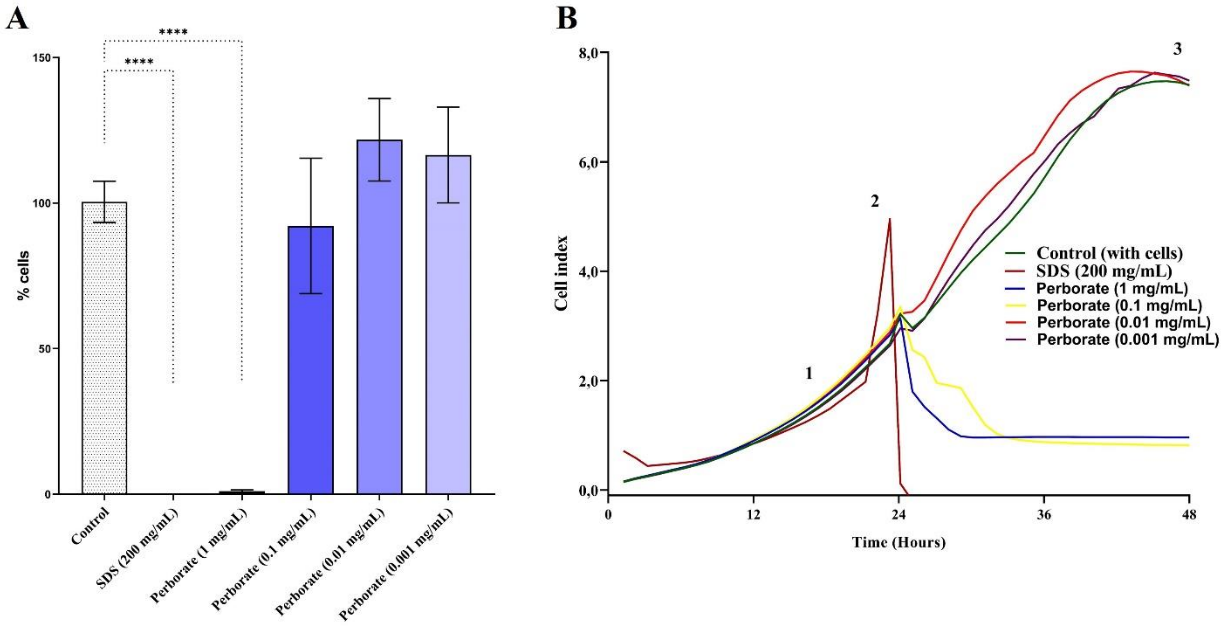

Supplementary Figure 3: Viability and Proliferation Assessment of HGF Cells in 2D Culture After Sodium Perborate Exposure. (A) MTT assay measuring cell viability after a 10 min exposure to varying concentrations of sodium perborate (1, 0.1, 0.01, 0.001 mg/mL) and 200 mg/mL SDS. Columns represent the mean ± 95% confidence interval (n=6). (B) Electrical impedance (xCELLigence) evaluation of cell

proliferation following a 10 min exposure to sodium perborate (n=3, triplicate). The graph highlights three phases: 1) Initial cell adherence and proliferation without treatment, 2) Proliferation following sodium perborate treatment, and 3) Plateau phase indicating stabilization. The basal medium served as the negative control, and SDS was used as a positive control for cell death. * (p < 0.05), ** (p < 0.01), *** (p < 0.001), **** (p < 0.0001).

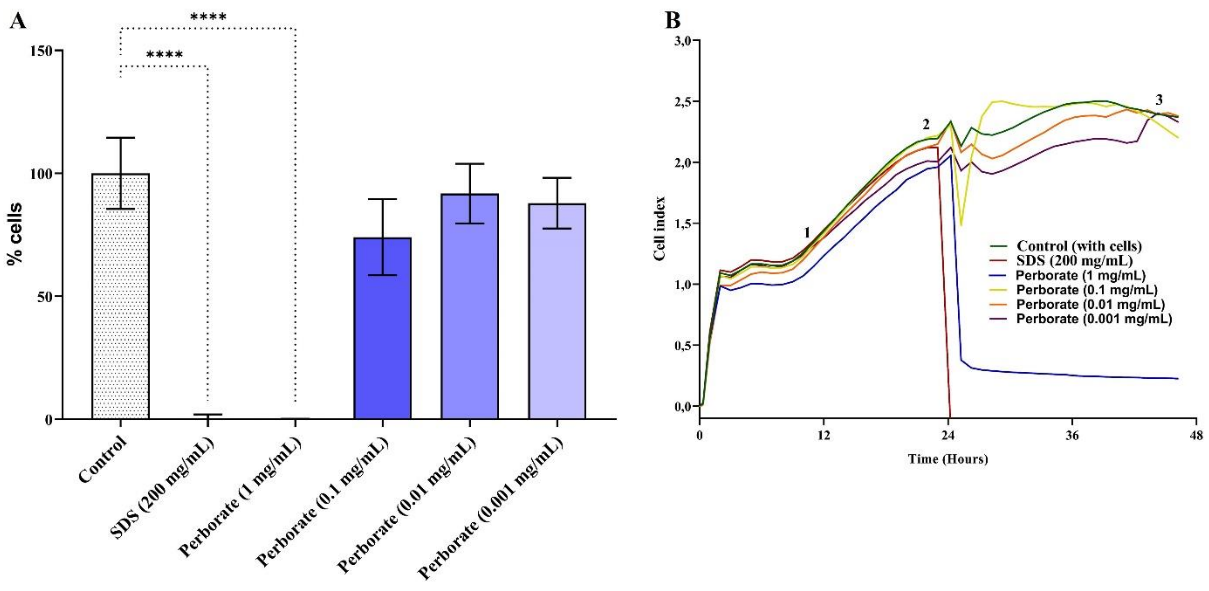

Supplementary Figure 4: Viability and Proliferation Assessment of HaCat Cells in 2D Culture After Sodium Perborate Exposure. (A) MTT assay illustrating cell viability after a 10 min exposure to varying concentrations of sodium perborate (1, 0.1, 0.01, 0.001 mg/mL) and 200 mg/mL SDS. Columns represent the mean ± 95% confidence interval (n=6). (B) Electrical impedance (xCELLigence) assessment of cell proliferation following a 10 min exposure to sodium perborate (n=3, triplicate). The graph highlights three phases: 1) Initial cell adherence and proliferation without treatment, 2) Proliferation after sodium perborate treatment, and 3) Plateau phase indicating stabilization. The basal medium served as the negative control, and SDS was used as a positive control for cell death. The graph represent the mean ± 95% confidence interval (n=6). * (p < 0.05), ** (p < 0.01), *** (p < 0.001), **** (p < 0.0001).

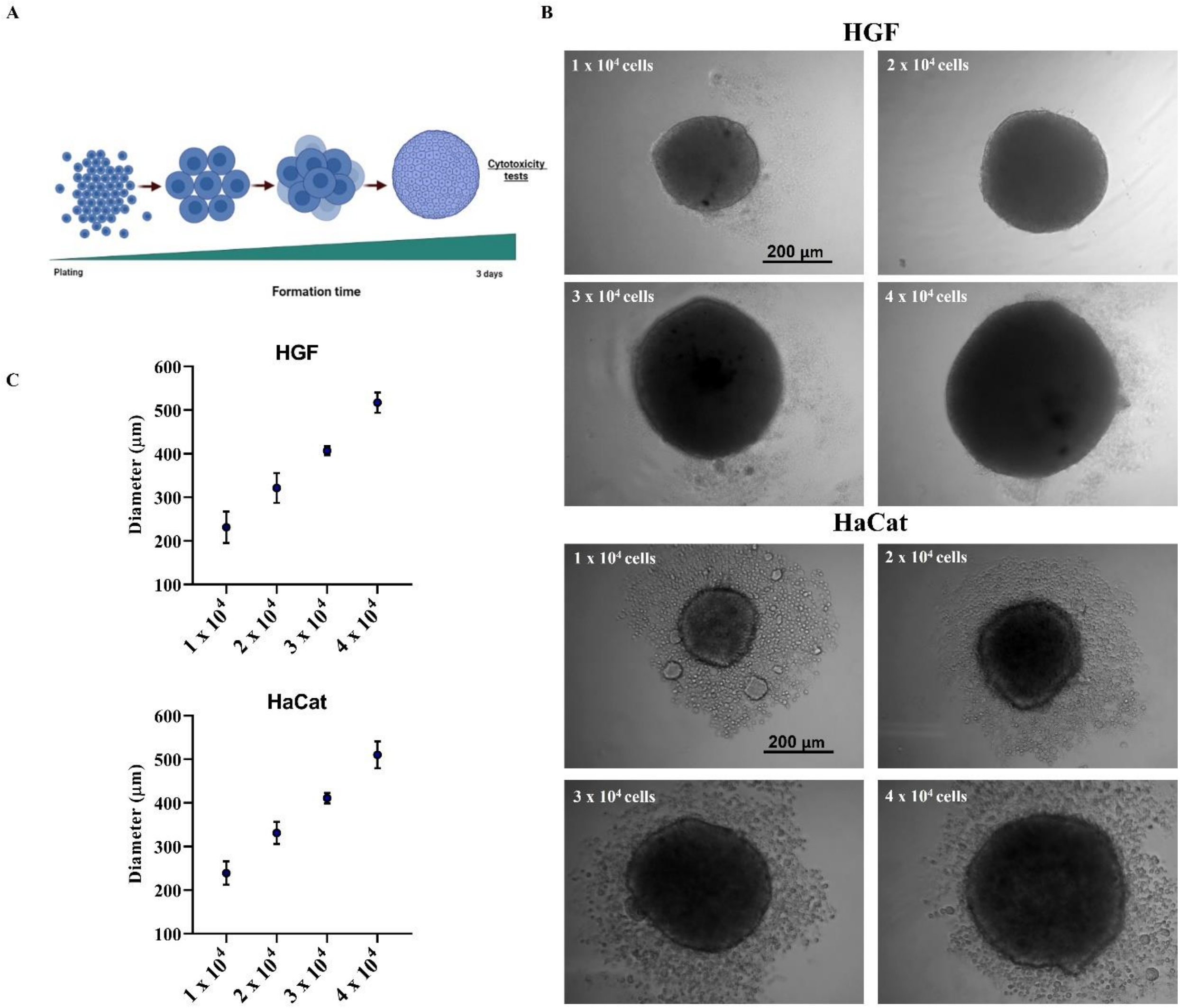

Supplementary Figure 5: Formation and Analysis of HGF and HaCat Spheroids. (A) Illustration of spheroid formation from the aggregation of suspended cells in non-adherent conditions. (B) Phase contrast microscopy images of spheroids formed at various cell densities (1 x 10^4, 2 x 10^4, 3 x 10^4, 4 x 10^4 cells/well). Scale bar = 200 µm. (C) Graph depicting the average spheroid diameter at different initial cell densities. The results in the graph are presented as mean values with a 95% confidence interval (n=24). Diameters were measured using phase contrast images and analyzed with J imaging software. All assays were conducted in triplicate.

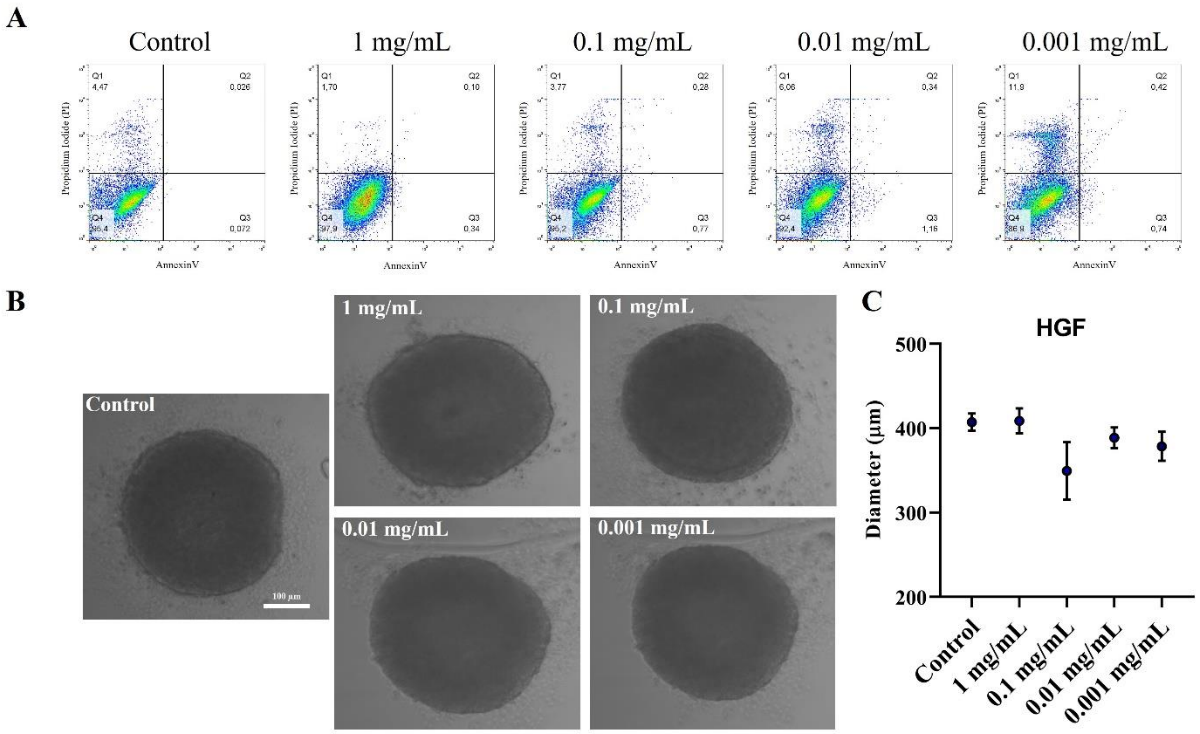

Supplementary Figure 6: Assessment of Viability, Morphology, and Diameter of HGF Spheroids Following Sodium Perborate Exposure. (A) Flow cytometry results for viability (Live and Dead) after exposure to varying concentrations of sodium perborate (1, 0.1, 0.01, 0.001 mg/mL). (B) Phase contrast microscopy images displaying HGF spheroid morphology after sodium perborate treatment. The basal medium served as the negative control. Scale bar = 100 µm. (C) Graph showing the average diameter of HGF spheroids after treatment with sodium perborate. The results in the graph are presented as mean values with a 95% confidence interval (n=24).

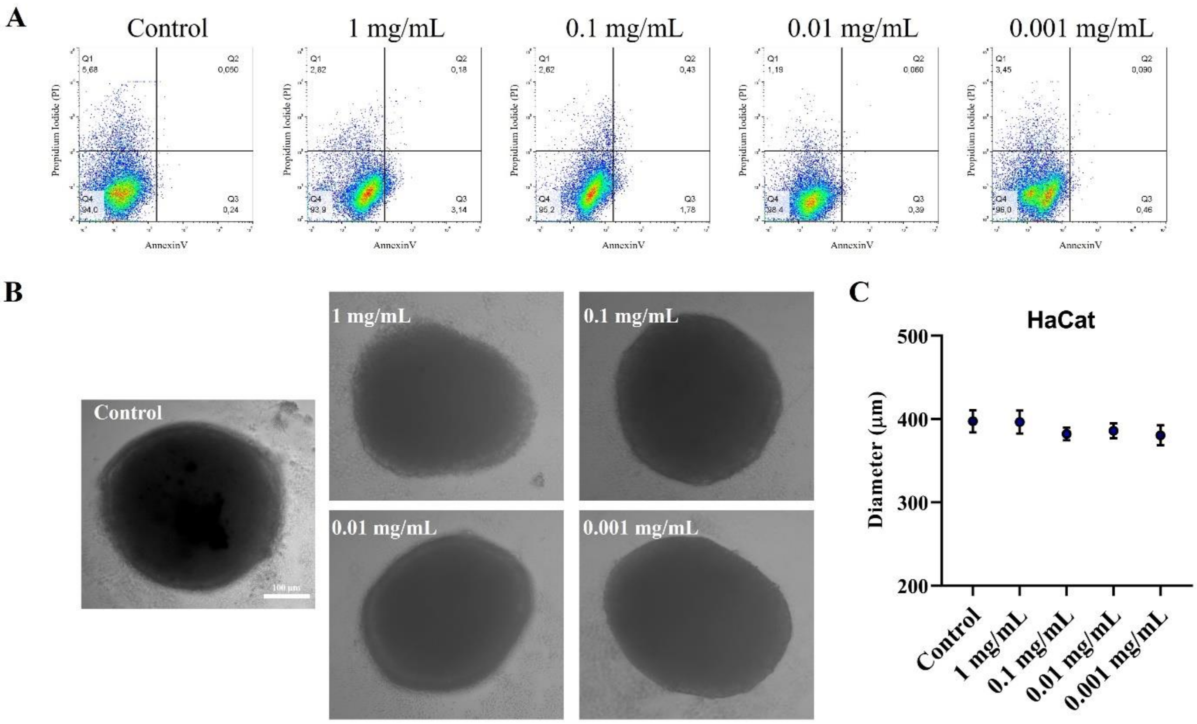

Supplementary Figure 7: Analysis of Viability, Morphology, and Diameter of HaCat Spheroids Following Sodium Perborate Exposure. (A) Flow cytometry results showing viability (Live and Dead) after exposure to varying concentrations of sodium perborate (0.001, 0.001, 0.1, 1 mg/mL). (B) Phase contrast microscopy images of HaCat spheroids following sodium perborate exposure. The basal medium served as the negative control. Scale bar = 100 µm. (C) Graph depicting the average diameter of HaCat spheroids after sodium perborate treatment. The results in the graph are presented as mean values with a 95% confidence interval (n=24).

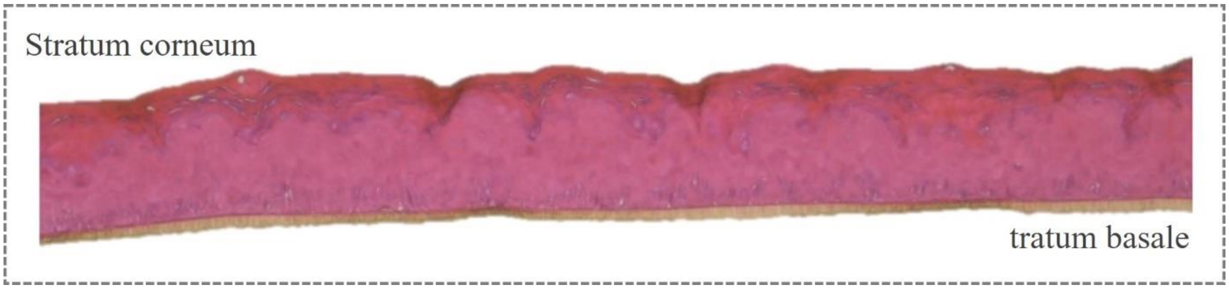

Supplementary Figure 8: Histology of the Reconstructed Epidermis Model. This histological figure was generated as an internal control for the EpiSkin™ cellular model before applying sodium perborate and other products. The stratum corneum and tratum basale are indicated in the figure.
